# Masitinib (AB1010), a Potent and Selective Tyrosine Kinase Inhibitor Targeting KIT

**DOI:** 10.1371/journal.pone.0007258

**Published:** 2009-09-30

**Authors:** Patrice Dubreuil, Sébastien Letard, Marco Ciufolini, Laurent Gros, Martine Humbert, Nathalie Castéran, Laurence Borge, Bérengère Hajem, Anne Lermet, Wolfgang Sippl, Edwige Voisset, Michel Arock, Christian Auclair, Phillip S. Leventhal, Colin D. Mansfield, Alain Moussy, Olivier Hermine

**Affiliations:** 1 INSERM, U891, Centre de Recherche en Cancérologie de Marseille, Signalisation, Hematopoiesis and Mechanisms of Oncogenesis, Centre de référence des mastocytoses, Marseille, France; 2 Institut Paoli-Calmettes, Marseille, France; 3 Univ Méditerranée, Marseille, France; 4 AB Science SA, Paris, France; 5 University of British Columbia, Department of Chemistry, Vancouver, British Columbia, Canada; 6 Institute of Pharmaceutical Chemistry, Martin-Luther-Universität, Halle, Wittenberg, Germany; 7 Laboratory of Oncology and Molecular Pharmacology, CNRS, UMR 8113, Ecole Normale Supérieure de Cachan, Cachan, France; 8 Service d'Hematologie, CNRS, UMR 8147, Centre de référence des mastocytoses, Université Paris V René Descartes, Hôpital Necker, Paris, France; Bauer Research Foundation, United States of America

## Abstract

**Background:**

The stem cell factor receptor, KIT, is a target for the treatment of cancer, mastocytosis, and inflammatory diseases. Here, we characterise the *in vitro* and *in vivo* profiles of masitinib (AB1010), a novel phenylaminothiazole-type tyrosine kinase inhibitor that targets KIT.

**Methodology/Principal Findings:**

*In vitro*, masitinib had greater activity and selectivity against KIT than imatinib, inhibiting recombinant human wild-type KIT with an half inhibitory concentration (IC_50_) of 200±40 nM and blocking stem cell factor-induced proliferation and KIT tyrosine phosphorylation with an IC_50_ of 150±80 nM in Ba/F3 cells expressing human or mouse wild-type KIT. Masitinib also potently inhibited recombinant PDGFR and the intracellular kinase Lyn, and to a lesser extent, fibroblast growth factor receptor 3. In contrast, masitinib demonstrated weak inhibition of ABL and c-Fms and was inactive against a variety of other tyrosine and serine/threonine kinases. This highly selective nature of masitinib suggests that it will exhibit a better safety profile than other tyrosine kinase inhibitors; indeed, masitinib-induced cardiotoxicity or genotoxicity has not been observed in animal studies. Molecular modelling and kinetic analysis suggest a different mode of binding than imatinib, and masitinib more strongly inhibited degranulation, cytokine production, and bone marrow mast cell migration than imatinib. Furthermore, masitinib potently inhibited human and murine KIT with activating mutations in the juxtamembrane domain. *In vivo*, masitinib blocked tumour growth in mice with subcutaneous grafts of Ba/F3 cells expressing a juxtamembrane KIT mutant.

**Conclusions:**

Masitinib is a potent and selective tyrosine kinase inhibitor targeting KIT that is active, orally bioavailable *in vivo*, and has low toxicity.

## Introduction

The stem cell factor (SCF) receptor, KIT, also called CD117 or c-KIT receptor, is a member of the type III receptor protein-tyrosine kinase family (RTK) [1]. This family also includes Flt3, the platelet-derived growth factor (PDGF) receptor, and the receptor for macrophage colony-stimulating factor/colony-stimulating factor-1 (c-Fms). SCF and KIT regulate erythropoiesis, lymphopoiesis, megakaryopoiesis, gametogenesis, melanogenesis, with SCF also serving as an important growth factor and activator of mast cells and eosinophils [1], [2]. It is known that SCF is up-regulated in inflammatory conditions and therefore presents a potential therapeutic target for the treatment of inflammatory diseases [3]. In addition, gain-of-function mutations in KIT, that is mutations that cause constitutive activation of the tyrosine kinase (TK), have been implicated in a variety of neoplasms including, gastrointestinal stromal tumours (GIST), mastocytosis, acute leukaemias, melanomas and other cancers [4], [5]. These mutations are concentrated in the fifth extracellular domain (exons 8 and 9), the juxtamembrane region (exon 11), and the kinase domain (exon 17) [6]. Also, autocrine or paracrine activation of KIT is thought to be involved in ovarian neoplasms and small-cell lung cancer [1], [6].

In the last decade, several inhibitors of TK have been developed for the treatment of cancer and other diseases. Imatinib mesylate (Gleevec, STI-571; Novartis, Basel, Switzerland) was the first TK inhibitor approved for clinical use [7]. This compound is a potent inhibitor of the PDGF receptor (PDGFR) [8] and also BCR-ABL, which causes chronic myelogenous leukaemia [9]. In addition, imatinib inhibits KIT, c-Fms and Syk [10], [11], and has been approved for the treatment of patients with KIT-positive nonresectable and/or malignant GIST. However, imatinib has a number of short-comings, including the development of resistance by most if not all patients with subsequent disease progression [12], as well as resistance of the D^816^V mutant, which is frequently associated with mastocytosis [6], [13], [14]. Moreover, imatinib may be cardiotoxic due to its inhibition of ABL [15], [16]. Therefore, novel TK inhibitors with improved selectivity are being developed for the treatment of diseases associated with KIT activation. Masitinib (AB1010), a protein TK developed by AB Science, S.A. (France), is one such new drug. The objective of this preclinical study was to provide a primary characterisation of the *in vitro* and *in vivo* activity of masitinib (mesylate salt) and to compare it against the benchmark protein TK inhibitor imatinib.

## Results

### Masitinib is an inhibitor of recombinant human KIT

**Figure 1:**
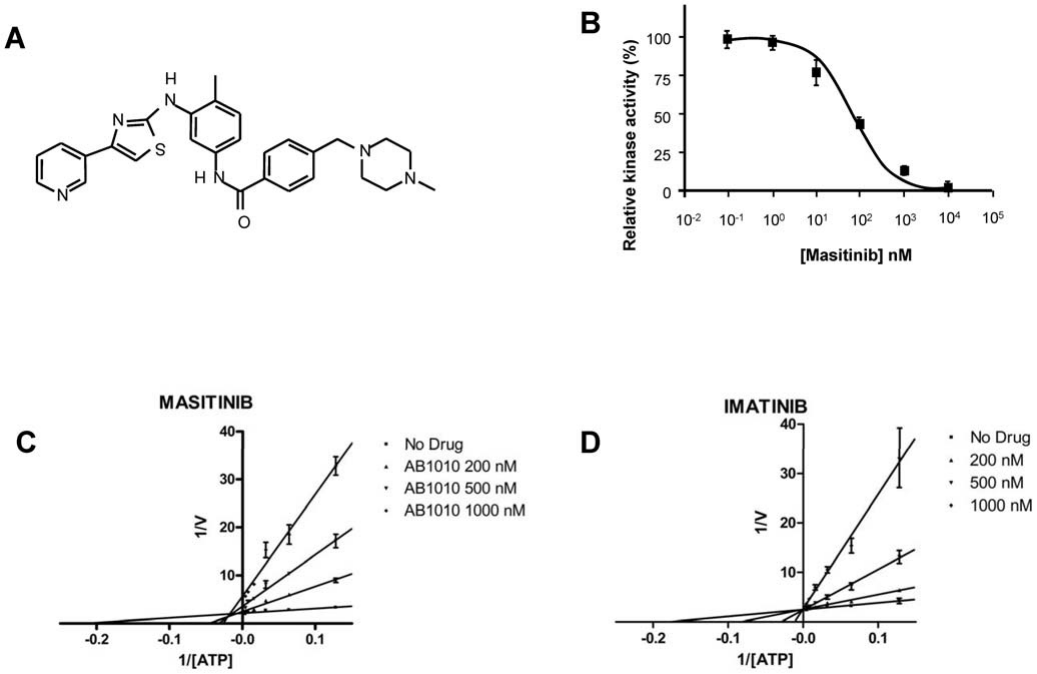
Masitinib inhibition of recombinant human KIT. (A) Structure of masitinib. The structure of masitinib is shown without its mesylate counterion. (B) Dose-response of masitinib at 10 µM ATP. Tyrosine phosphorylation by KIT was assayed by measuring the incorporation of phosphate into poly(Glu,Tyr 4∶1). Lineweaver-Burk Plots for masitinib (C) and imatinib (D) with ATP as the varied substrate. Recombinant human KIT tyrosine kinase assays were performed using an ELISA-based assay with poly(Glu,Tyr 4∶1) as a substrate. In (C), the lines intersect to the left of the Y-axis, indicating a mixed mechanism of inhibition for masitinib, whereas in (D), the lines intersect on the Y-axis, indicating a competitive mechanism of inhibition for imatinib.

Activity of the synthetic TK inhibitor masitinib (mesylate salt; Figure 1A) was assessed using a recombinant human wild-type KIT protein corresponding to the intracellular domain (amino acids 567–976). Using poly(Glu,Tyr 4∶1) as a substrate, the recombinant protein had a K_m_ for ATP of 9.0±2.0 µM (data not shown). Masitinib inhibited the recombinant enzyme with a half inhibitory concentration (IC_50_) of 200±40 nM (Table 1 and Figure 1B). Kinetic studies in which ATP and masitinib were covaried showed that at concentrations ≤500 nM masitinib is a competitive inhibitor against ATP, but at higher concentrations (>1 µM), it has a mixed mechanism of inhibition against ATP (Figure 1C). Under identical assay conditions and with the same enzyme, imatinib had an IC_50_ of 470±120 nM (see Supporting Information; Table S1) and was a strictly competitive inhibitor against ATP (Figure 1D).

**Table 1:** Effect of masitinib on the activity of protein kinases.

Protein kinase	Recombinant enzyme IC_50_ (µM)	Cell-based assay IC_50_ (µM)
*Class III receptor tyrosine kinases*		
KIT wild-type	0.20±0.04	0.15±0.08
KIT V^559^D	-	0.003±0.0001
KIT D^816^V	>10	5.0±2.0
KIT D^814^V (murine)	-	3.0±0.1
KIT Δ27 (murine)	-	0.005±0.0003
PDGFRβ	0.80±0.12	0.05±0.02
PDGFRα	0.54±0.06	0.3±0.005
Flt3	>10	5.0±2.0
c-Fms	1.48±0.54	1.0±0.03
*Other receptor tyrosine kinases*		
VEGFR1	>10	-
VEGFR2	>10	-
Epidermal growth factor receptor	>10	7.0±0.8
Fibroblast growth factor receptor 1	>10	7.0±1.9
Fibroblast growth factor receptor 3	>10	5.5±2.8
Insulin-like growth factor-I receptor	>10*	10.0±0.67
c-Met	>10	-
TrkB	-	7.0±1.9
c-Ret	>10	8.0±1.2
Alk	-	9.0±0.18
*Nonreceptor tyrosine kinases*		
ABL1	1.20±0.34	2.8±0.8
Focal adhesion kinase	>10	-
Proline-rich Tyrosine kinase (FAK2/PYK2)	>10	-
Lyn B	0.51±0.13	-
Src	1.87±0.31	-
Hck	2.0±0.2	-
Jak1	-	8.0±1.4
Jak2	>10*	10.0±0.8
Jak3	-	10.0±0.5
Tyk2	-	9.0±0.8
Btk	>10	-
Bmx	>10	-
Syk	>10	-
Fes	>10	
*Serine/threonine kinases*		
Protein kinase C-α	>10*	-
Pim1	>10*	-
Akt1	>10*	-

Recombinant tyrosine kinase assays were performed using an ELISA-based assay with poly(Glu,Tyr, 4∶1) as the substrate. All protein kinases were human versions except where noted. Cell-based assays were performed using Ba/F3 cells expressing the various enzymes, and cell proliferation was assessed using WST-1. All concentrations were tested in duplicate (recombinant assays) or triplicate (cell-based assays). Results are the means±standard deviations from at least three independent experiments except where noted.

* Results are from a single experiment performed as part of a kinase screen by Proqinase (Germany).

### Masitinib inhibits human and murine KIT in intact cells

**Figure 2:**
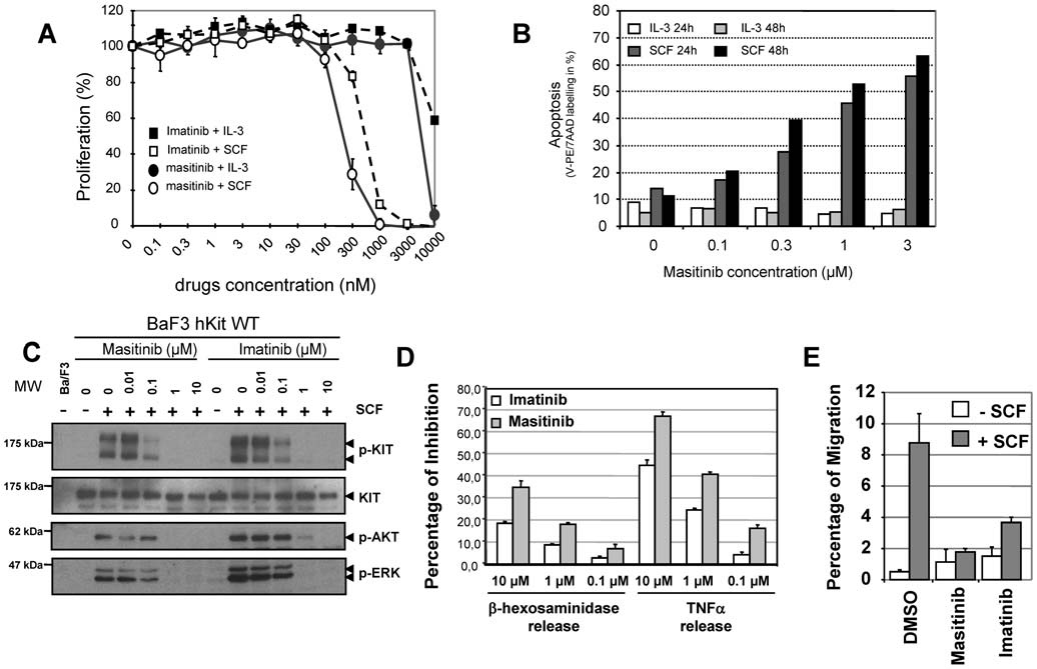
Masitinib inhibition of KIT in intact cells. (A) Effect of masitinib and imatinib on SCF and IL-3-stimulated cell proliferation. Ba/F3 cells expressing wild-type (WT) human (hKIT) were incubated for 48 hours with 0.1% conditioned medium from X63-IL-3 cells (IL-3) (filled symbols) or 250 ng/ml murine SCF in the presence of various concentrations of masitinib and imatinib. Cell proliferation was assessed by WST-1 colorimetric assay. (B) Induction of apoptosis by masitinib in Ba/F3 cells expressing wild-type human KIT. Cells were incubated for 24 hours with stem cell factor (SCF) or 0.1% conditioned medium from X63-IL-3 cells (IL-3) in the presence of various concentrations of masitinib. Apoptosis was assessed via Annexin V-phycoerythrin (PE) and 7-amino-actinomycin D (7-AAD) staining, followed by fluorescence-activated cell sorting. A second dataset was acquired for an incubation of 48 hours to verify completeness of the apoptosis process. (C) Effect of masitinib and imatinib on KIT tyrosine phosphorylation in Ba/F3 cells (upper panels) and phosphorylation of the downstream targets AKT and ERK (lower panels). Ba/F3 cells expressing wild-type human KIT (hKIT WT) were incubated for 5 minutes with (+) or without (-) 250 ng/ml murine SCF in the presence of various concentrations of masitinib and imatinib. Tyrosine phosphorylation of KIT, AKT and ERK, were assessed by immunoprecipitation (IP) with the relevant antibody, followed by western blotting (Blot) with anti-phosphotyrosine (pTyr) or anti-KIT molecular weight. Results are representative of at least three independent experiments. MW = molecular weight markers. (D) Comparison of masitinib's and imatinib's ability to inhibit the FcεRI-mediated degranulation and cytokine production in cord blood derived mast cells (CBMC). Left: effect on the release of β-hexosaminidase by IgE-anti IgE activated CBMC after 30 minutes of stimulation. Right: effect on cytokine production by IgE-anti IgE-activated CBMC after 4 hours of simulation via ELISA assessment of TNF-α release. (E) The effect of masitinib and imatinib on the migration of murine BMMCs in response to rmSCF stimulation.

Assessment of masitinib's and imatinib's ability to inhibit the function and activity of KIT in cells was conducted using the interleukin-3 (IL-3)-dependent cell line, Ba/F3 [17]. These cells normally cannot survive in the absence of IL-3, but they proliferate when transfected with transforming mutants of TKs or when transfected with wild-type receptor TKs and treated with the appropriate growth factor. In Ba/F3 cells expressing human wild-type KIT, masitinib dose-dependently inhibited SCF-induced cell proliferation with an IC_50_ of 150±80 nM, (Table 1 and Figure 2A). In contrast, the IC_50_ for inhibition of IL-3-stimulated proliferation occurred at approximately >5 µM, with inhibition in this case due to the ability of high concentrations of masitinib to inhibit other TKs in the cells. Imatinib showed a similar inhibitory pattern in this proliferation assay. Fluorescence-activated cell sorting (FACS) analysis of Annexin V/7-amino-actinomycin D-stained cells revealed that masitinib causes a dose-dependent induction of apoptosis in SCF-treated Ba/F3 cells expressing wild-type human KIT (Figure 2B). In contrast, masitinib-treated cells were rescued from apoptosis when treated with IL-3. Qualitative analyses by immunoprecipitation-western blotting experiments revealed that masitinib caused a parallel inhibition of SCF-stimulated tyrosine phosphorylation of human KIT, which was again observed with imatinib (Figure 2C). Inhibition of the KIT receptor was also associated with a parallel inhibition of KIT-secondary messengers such as AKT and ERK activation, with comparable dose effects observed between masitinib and imatinib treatment.

### Masitinib inhibits human mast cell degranulation, cytokine production and migration of bone marrow cells

Assessment of masitinib's and imatinib's ability to inhibit the FcεRI-mediated degranulation of human cord-blood-derived mast cells (CBMC) showed that both compounds produced a dose-dependent inhibition β-hexosaminidase release by IgE-anti IgE activated CBMC after 30 minutes of stimulation (Figure 2D left). At concentrations of up to 10 µM, neither compound was able to completely block the release of this mediator; however, although not statistically different (p = 0.1), masitinib tended to be more potent than imatinib. At concentrations of 10, 1.0 and 0.1 µM, imatinib only slightly inhibited β-hexosaminidase release by 19, 8 and 2%, respectively, compared to an inhibition of 35, 18 and 7%, respectively for masitinib. This effect was not due to cytotoxicity, as evident from the incubation of CBMC with masitinib for up to 9 hours having no affect on cell viability. Also, a possible confounding effect associated with the vehicle used to deliver masitinib or imatinib dimethyl sulphoxide (DMSO) can be excluded because the concentration used was below the threshold of effect.

The effect of masitinib and imatinib on cytokine production of IgE-anti IgE-activated CBMC was explored via ELISA assessment of TNF-α release. As shown in the right panel of Figure 2D, masitinib and imatinib dose-dependently inhibited the release of TNF-α after 4 hours of stimulation. At concentrations of 10, 1.0 and 0.1 µM, masitinib inhibited TNF-α release by 68, 40 and 16%, respectively, whereas imatinib resulted in a weaker inhibition (p = 0.1) of 45, 24 and 4%, respectively. Hence, neither compound was able to completely block the release of this mediator, although both more potently inhibited TNF-α release than β-hexosaminidase release.

The KIT receptor is involved in mast cell migration [18]. We assessed the effect of masitinib and imatinib on murine bone marrow mast cell (BMMC) migration in response to recombinant mouse stem cell factor (rmSCF) stimulation (Figure 2E). After 4 hours of stimulation in the absence of either inhibitor, we observed a migration of BMMCs in response to SCF compared to unstimulated BMMCs (average migration of 8.7% versus 0.5% of the initial concentration, respectively). Upon treatment with 1.0 µM of masitinib, migration of SCF-stimulated BMMCs was inhibited approximately79.6% (p = 0.029) relative to the control. Imatinib similarly inhibited SCF-stimulated BMMC migration (58.1% relative to control), although this inhibition was significantly weaker than that of masitinib (p = 0.029).

### Masitinib inhibits KIT gain-of-function mutants

**Figure 3:**
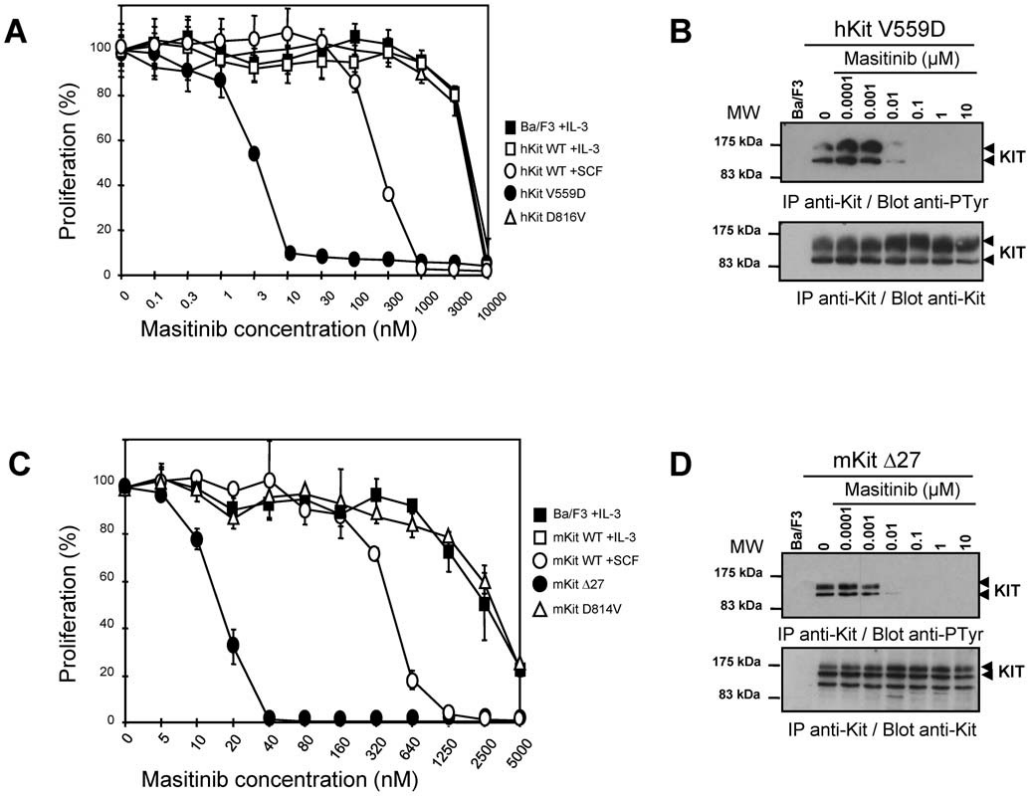
Effect of masitinib on human and mouse KIT mutants. Effect of masitinib on the proliferation of Ba/F3 cells expressing wild-type (WT) or mutant human (hKIT) (Fig. 3A) or murine (Fig. 3C) KIT (mKIT). Assessment of proliferation was as described for Fig. 2A. Effect of masitinib on tyrosine phosphorylation of KIT mutants in Ba/F3 cells expressing the human V^559^D mutant (hKIT V559D) (Fig. 3B) or murine Δ27 mutant (mKIT Δ27) (Fig. 3D). KIT tyrosine phosphorylation was assessed as described in Fig. 2B. IP = immunoprecipitation; Blot = western blot; MW = molecular weight markers.

Gain-of-function mutations in KIT are associated with mastocytosis, GIST, and various human neoplasms [6]. In Ba/F3 cells, masitinib dose-dependently inhibited cell proliferation induced by the V^559^D mutant, commonly associated with GIST (exon 11), with an IC_50_ of 3.0±0.1 nM (Figure 3A and Table 1). Masitinib also caused a parallel inhibition of the tyrosine phosphorylation of this mutant (Figure 3B). In the Δ27 mouse mutant of KIT, which has a deletion of codons 547–555 in the juxtamembrane domain (exon 11) known to cause constitutive activation and ligand-independent cell proliferation [19], masitinib dose-dependently inhibited Δ27 KIT-dependent proliferation of Ba/F3 cells with an IC_50_ of 5.0±0.3 nM (Table 1 and Figure 3C). Masitinib also caused a parallel reduction in its tyrosine phosphorylation (Figure 3D). In contrast, masitinib only weakly inhibited the proliferation of Ba/F3 cells expressing the D^816^V mutant of KIT, which is associated with adult mastocytosis and myeloproliferative disorder-acute myeloid leukaemia (exon 17), with an IC_50_ of 5.0±2.0 µM (Figure 3A and Table 1). This result was corroborated by assays using recombinant human KIT intracellular domain with the D^816^V mutation (Table 1) and its murine equivalent D^814^V mutant, for which masitinib had an IC_50_ of 3.0±0.1 µM (Figure 3C and Table 1).

**Figure 4:**
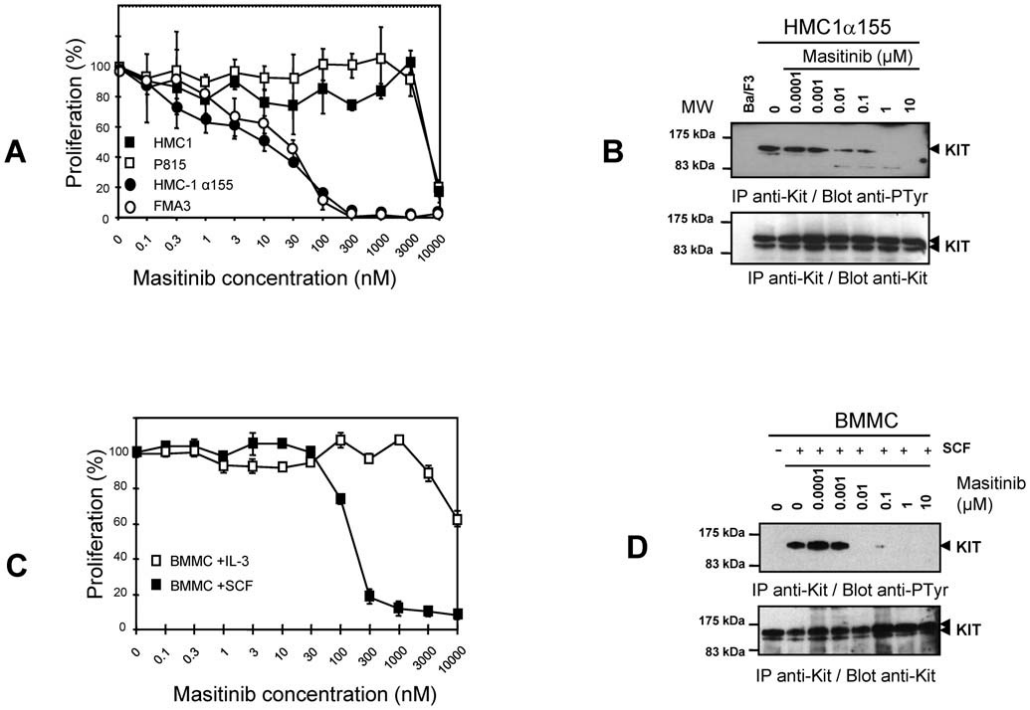
Effect of masitinib on cell proliferation and KIT tyrosine phosphorylation in mastocytoma cell-lines and BMMC. (A) Effect of masitinib on the proliferation of human (HMC1, HMC-1α155) (filled symbols) and murine (P815, FMA3) mastocytoma cell lines harboring KIT mutants. Cells were incubated for 2 days with the indicated concentrations of masitinib. (B) western blotting analysis of HMC-1α155 tyrosine phosphorylation. (C) Effect of masitinib in the proliferation of BMMCs. BMMCs were incubated for 2 days with 250 ng/ml of stem cell factor (SCF) or 0.1% conditioned medium from X63-IL-3 cells (IL-3) with the indicated concentrations of masitinib. (D) Western blotting analysis of BMMC tyrosine phosphorylation. Cell proliferation was assessed by WST-1 colorimetric assay. Tyrosine phosphorylation of the KIT protein from sensitive cell types in (A) and (C) was analysed by immunoprecipitation (IP) and examined by western blotting (Blot) with antibodies to phosphotyrosine (anti-pTyr) or KIT (anti-Kit). MW = molecular weight.

To confirm the results in Ba/F3 cells, masitinib was tested in various mastocytoma cell lines. In HMC-1α155 and FMA3 cells, which carry KIT with mutations in the juxtamembrane domain [20], the IC_50_ values were approximately 10±1 nM and 30±1.5 nM, respectively (Figure 4A). Immunoprecipitation-western blotting experiments on HMC-1α155 revealed parallel reductions in KIT tyrosine phosphorylation (Figure 4B). Finally, the effect of masitinib on primary BMMCs from mice expressing wild-type KIT was examined. Masitinib inhibited SCF-stimulated cell proliferation (Figure 4C) and tyrosine phosphorylation of KIT (Figure 4D) with an IC_50_ of 200±50 nM, whereas the IC_50_ for IL-3-stimulated proliferation in these cells was >10 µM (Figure 4C).

### Selectivity of masitinib

**Figure 5:**
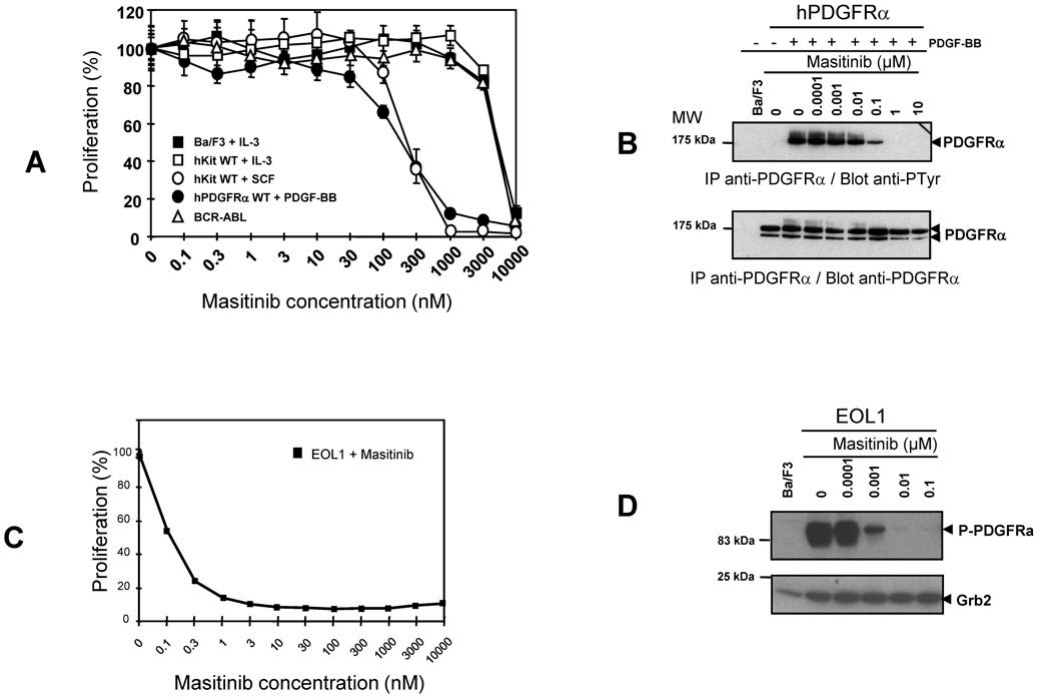
Effect of masitinib on BCR-ABL and PDGFRα. (A) Effect of masitinib on the proliferation of Ba/F3 cells expressing human wild-type KIT (hKIT WT), BCR-ABL, human wild-type PDGFRα (hPDGFRα WT). Cells were treated for 48 hours with PDGF-BB, IL-3, or SCF and in the presence of various concentrations of masitinib. Cell growth was assessed by WST-1 colorimetric assay. (B) Ba/F3 cells expressing hPDGFRα were treated for 5 minutes with PDGF-BB and various concentrations of masitinib. Tyrosine phosphorylation of PDGFRα was analysed by immunoprecipitation (IP), followed by western blotting (Blot) with an anti-phosphotyrosine (pTyr) antibody (upper panel) and an anti-PDGFRα antibody (lower panel). Results are representative of two independent experiments. (C) Effect of masitinib on the proliferation of EOL1 cells, a hyperoesinophilic tumour cell line expressing the FIP1L1-PDGFRα chimeric protein. (D) Western blotting analysis of EOL1 tyrosine phosphorylation. MW = molecular weight markers.

Many TK inhibitors targeting KIT additionally inhibit other members of the class III TK receptors, especially ABL and PDGFRs [8], [9], [14], [21]. A study of masitinib's inhibitory action on a selection of these TKs was therefore conducted (Figure 5A and Table 1), along with a parallel examination of imatinib for direct comparison of their IC_50_ values (see Supporting Information; Table S1). In Ba/F3 cells expressing PDGFR-α, masitinib inhibited PDGF-BB-stimulated proliferation and PDGFR-α tyrosine phosphorylation (Figure 5B) with an IC_50_ of 300±5 nM. In contrast, masitinib showed relatively weak inhibition of cell proliferation in Ba/F3 cells expressing BCR-ABL, with an IC_50_ of 2800±800 nM. The corresponding recombinant assays show that masitinib inhibits the *in vitro* protein kinase activity of PDGFR-α and β with IC_50_ values of 540±60 nM and 800±120 nM, respectively, and to a lesser extent ABL1, with an IC_50_ of 1200±300 nM (Table 1). Comparatively, imatinib inhibits the *in vitro* protein kinase activity of PDGFR-α, PDGFR-β and ABL1 with IC_50_ values of 400 nM, 440±120 nM, and 270±130 nM, respectively (see Supporting Information; Table S1). Against other class III RTK, masitinib was inactive against Flt3 (>10 µM) but moderately inhibited c-Fms in both cell proliferation and recombinant protein kinase assays (IC_50_ of 1.0±0.03 µM and 1.48±0.54 µM, respectively). In addition, strong inhibition of proliferation was observed in EOL1 cells (IC_50_ of 0.2±0.1 nM; Figures 5C), a hypereosinophilic tumour cell line expressing the FIP1L1-PDGFRα chimeric protein, which is associated with chronic eosinophilic leukaemia. Similar inhibition was observed for tyrosine phosphorylation of the FIP1L1-PDGFRα chimeric protein (Figures 5D). This is a factor of 10^3^ lower than that for the wild-type PDGFRα receptor.

To extend the range of protein kinases tested against masitinib, various receptor TKs (VEGFR1 & 2; epidermal growth factor receptor; fibroblast growth factor receptor 1 & 2; insulin-like growth factor-I receptor; c-Met; TrkB; and c-Ret) and nonreceptor TKs (focal adhesion kinase; Lyn B; Src; Hck; Jak1; Jak2; Jak3; Tyk2; Btk; Bmx; and Syk) were examined using both recombinant and cell-based assays (Table 1). In general, masitinib was found to be either inactive or a weak inhibitor of all these TKs, with the exception of recombinant Lyn B, for which the IC_50_ was 510±130 nM. Finally, masitinib was inactive against three recombinant serine/threonine kinases (protein kinase C-α, Akt1, and Pim-1).

### Molecular modelling of masitinib binding to KIT and ABL

**Figure 6:**
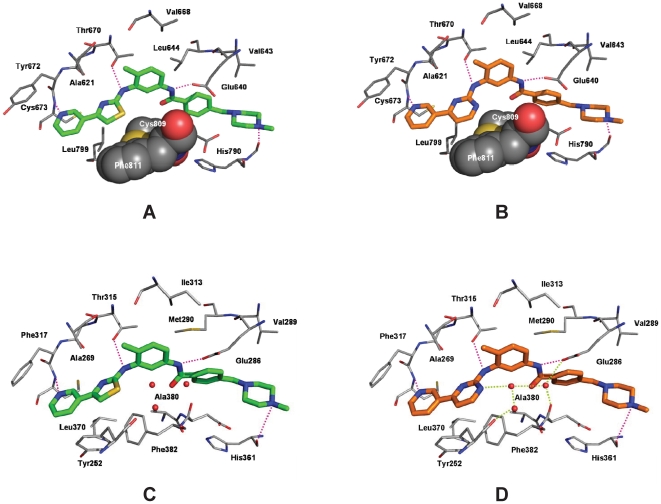
Docking of masitinib to human KIT and ABL: comparison with imatinib binding. (A and B) details of the binding of masitinib (A; green; docking pose) and imatinib (B; orange; X-ray structure 1T46.pdb) to the KIT kinase domain. Masitinib and imatinib interact with the protein via hydrogen bonds involving Glu640, Thr670, Cys673, and His790 and van der Waals interactions with Ala621, Val643, Leu644, Val668, Tyr672, Leu799, Cys809, and Phe811. Cys809 and Phe811, which form a hydrophobic groove for the thiazole and pyrimidine ring, respectively, are shown as space-filling structures. (C and D) Details of the binding of masitinib (C; green; docking pose) and imatinib (right site; orange; X-ray structure 1IEP.pdb) to the ABL kinase domain. Masitinib and imatinib interact with the protein via hydrogen bonds involving Glu286, Thr315, Phe317, and His361 and van der Waals interactions with Tyr252, Ala268, Val289, Met290, Ile313, Phe317, Leu370, and Phe382. In addition, the pyrimidine ring of imatinib is involved in a hydrogen bond network to conserved water molecules around the DFG motif of ABL (shown as red balls).

Molecular modelling studies were performed to help determine how masitinib binds selectively to KIT and to compare its mode of binding to that of imatinib (Figure 6). Masitinib was docked into the ATP-binding site of wild-type KIT and ABL using the coordinates of human KIT and ABL in the inactive conformation. Both kinases have been co-crystallised with imatinib (STI-571) [22], [23]. When docked into the KIT binding site, the aminothiazole of masitinib participates in a hydrogen bond with the side-chain of the gatekeeper residue Thr670. The amide NH forms a hydrogen bond to the side-chain of Glu640, and the *meta*-nitrogen of the pyridine ring interacts with the backbone NH of Cys673 (Figure 6A). For the methylpiperazine group, an additional hydrogen bond is observed between the protonated CH_3_-NH and the backbone-CO of His790. The thiazole ring of masitinib packs loosely between the aliphatic portions of the side-chains of Ala621, Leu799, Cys809, and Phe811. Binding of masitinib to ABL occurs in a similar manner, although small differences are observed near the DFG motif (Phe810 in KIT and Phe382 in ABL) (Figure 6C). There are close similarities between the modes of KIT and ABL binding for imatinib and masitinib (Figures 6B and 6D). Differences are apparent, however, in the ABL complex (Figure 6D), where the polar pyrimidine ring of imatinib is involved in a strong hydrogen bond network to three co-crystallised water molecules bound to the DFG motif. In the KIT-imatinib X-ray structure (Figure 6B), only one loosely bound water molecule is observed in the corresponding region indicating a more hydrophobic environment. This dissimilarity arises because the thiazole ring of masitinib is more hydrophobic than imatinib's pyrimidine ring and is unable to mediate a hydrogen bond to the water molecules. Consequently, preferred binding of masitinib by KIT is observed.

### Masitinib inhibits tumour growth *in vivo*

**Figure 7:**
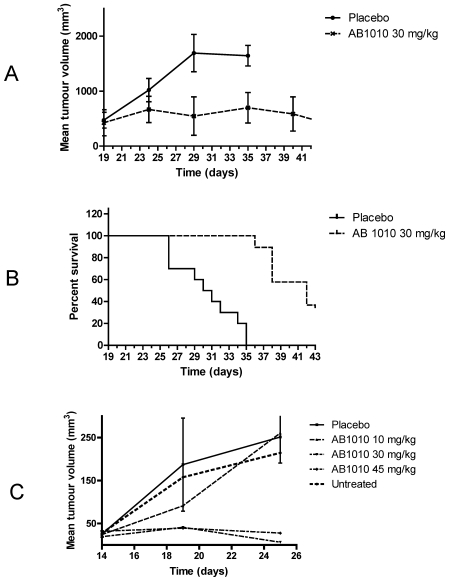
Masitinib inhibits tumour growth *in vivo*. Nude mice were gamma-irradiated and after 24 hours, injected subcutaneously with 1.5×10^6^ Ba/F3 cells expressing the murine Δ27 KIT mutant. (A and B) Effect of intraperitoneal administered masitinib treatment on Δ27 KIT-expressing tumours, with an average pre-treatment tumour volume of 400 mm^3^ (large tumour experiment). Mice were treated with 30 mg/kg masitinib or a placebo (vehicle control) (n = 10 per group) twice daily for 25 days by intraperitoneal injection. (A) Mean tumour volume assessed every 5 days during the treatment. D19 corresponds to the first day of treatment. (B) Kaplan-Meier survival plot. (C) Effect of oral masitinib treatment on Δ27 KIT-expressing tumours, with an average pre-treatment tumour volume of 40 mm^3^ (small tumour experiment). Mice were treated twice daily for 11 days with masitinib administered orally at 0 (controls), 10, 30, or 45 mg/kg. D14 corresponds to first day of treatment.

A mouse model of tumour growth with Δ27-expressing Ba/F3 cells was used to investigate masitinib's *in vivo* activity. Nude mice were gamma-irradiated and implanted after 24 hours with Δ27-expressing Ba/F3 cells by subcutaneous injection. When the tumours had grown to an average volume of 400 mm^3^ (19 days post implantation of tumour cells), mice were treated with intraperitoneal injection of 30 mg/kg masitinib or placebo (i.e. vehicle control) (n = 10 per group) twice daily for 25 days and tumour volume was assessed every 5 days. At the start of treatment, the mean tumour volumes were not statistically different between groups (p = 0.617). Tumour growth stabilised in mice treated with masitinib, whereas placebo treated mice had a mean doubling time of 5 days, (Figure 7A). A significant difference in average tumour volume was evident after 10 days of treatment (day 29), the placebo group showing an approximate 4-fold increase compared to the masitinib treated group (p = 0.016). The administered dose of masitinib did not affect the total body weight of the mice during the course of the study. Furthermore, as shown in Figure 7B, masitinib increased the median survival time from 30.5 to 42 days (p<0.001) relative to the control population.

To examine the effect of orally administered masitinib on small tumour volumes, mice with an average tumour volume of 40 mm^3^ (14 days post implantation of tumour cells) were assigned to one of five groups: masitinib at 10, 30, or 45 mg/kg; placebo (vehicle control); or untreated (n = 8 per group). At the start of treatment, the mean tumour volumes were not statistically different between groups (p = 0.236). Treatment was administered twice daily for 10 days with tumour size measured every 5 days during the treatment period. Mice treated with masitinib showed a dose-dependent inhibition of tumour growth, whereas the vehicle-treated population showed continuous tumour growth with an estimated doubling time of 1 day; corresponding to a tumour volume increase of 1200% between days 14 to 25 (Figure 7C). Masitinib at 30 or 45 mg/kg significantly reduced tumour growth following 11 days of treatment compared to placebo, with average tumour volume increases of 355% (p = 0.05) and 154% (p = 0.005), respectively in the masitinib-treated mice. However, the lower masitinib dose of 10 mg/kg did not substantially alter tumour size relative to control (p = 0.940). For one and two animals receiving masitinib at 30 and 45 mg/kg respectively, there were no detectable tumours at day 25. These doses of masitinib did not affect body weight gain of the mice during the course of the study. Finally, we performed a separate experiment to examine the effect of twice daily, orally administered masitinib at 100 mg/kg on mice having large Δ27 KIT-expressing tumours (average tumour volume 500 mm^3^, 26 days post implantation of tumour cells). We found that tumour growth was blocked following 5 days of treatment with masitinib (data not shown). Upon withdrawal of masitinib treatment after day 5, tumour growth was once again evident.

## Discussion

In the current set of experiments we have characterised the *in vitro* and *in vivo* profiles of masitinib, a novel phenylaminothiazole-type TK inhibitor. Of the protein kinases tested, the most sensitive to masitinib were KIT and PDGFR, both of which had submicromolar IC_50_ values. In addition, masitinib was a good inhibitor of Lyn kinase (IC_50_ of 510±130 nM compared to 2200±100 nM for imatinib, see Supporting Information; Table S1), and to a lesser extent, fibroblast growth factor receptor 3. In contrast to many other KIT inhibitors, such as imatinib, masitinib is a relatively weak inhibitor of ABL (IC_50_ for recombinant KIT = 1.2±0.34 µM for masitinib versus0.27±0.13 µM for imatinib), and the relative selectivity for KIT versus ABL was 10-fold higher for masitinib than for imatinib (ABL IC_50_/KIT IC_50_ = 6.0 for masitinib versus 0.6 for imatanib). Masitinib was shown to be inactive against Flt3 and a relatively weak inhibitor of c-Fms, which are two members of the class III RTKs. Masitinib was also inactive against the vascular endothelial growth factor receptor, a RTK often inhibited by KIT inhibitors [21], [24], [25]. In contrast, other KIT inhibitors, including imatinib, dasatinib (Sprycel, Bristol-Myers Squibb), and sunitinib (Sutent, Pfizer), also inhibit several other protein kinases, especially other members of the type III receptor TK family [21], [26]. Thus, masitinib appears to be the most specific inhibitor of KIT. Our molecular modelling studies suggest that this greater selectivity of masitinib may be due to an inability to form hydrogen bonds to three water molecules in the active site of ABL, despite both compounds binding to the active sites of KIT and ABL with similar conformations [22].

The lack of specificity associated with other KIT inhibitors may lead to toxic side effects and recent studies suggest that imatinib may be cardiotoxic due to inhibition of ABL [15], [16]. Indeed, the cardiotoxicity of imatinib was reported with observation of left ventricular dysfunction and even frank congestive heart failure in patients without a prior history of heart disease [15]. In contrast, the pharmacological profile of masitinib shows that it does not target the kinases presumably involved in cardiotoxicity, e.g. SRC, vascular endothelial growth factor receptors (VEGFR), endothelial growth factor receptors (EGFR) and Abelson proto-oncogene ABL. Thus, the risk of cardiotoxicity appears to be lower with masitinib than with imatinib. In addition to cardiotoxicity, imatinib has been shown to be genotoxic as indicated by a positive chromosome aberration test in human lymphocytes in Chinese Hamster Ovary (CHO) cells and in a bacterial reverse mutation test [27]. Masitinib, in contrast, is not mutagenic in bacterial reverse mutation tests using *Salmonella typhimurium* and *Escherichia coli* and does not cause chromosome aberrations in cultured human lymphocytes. Masitinib also does not cause damage to chromosomes or the mitotic apparatus in mouse bone marrow cells following two daily administrations at 437.5, 875, or 1750 mg/kg/day, and it is not mutagenic in a mouse lymphoma assay (our unpublished results).

Importantly, masitinib was a potent inhibitor of several gain-of-function KIT mutants, including V^559^D (exon 11), which is associated with GIST [6], and a murine KIT mutant with a deletion of nine amino acids in the juxtamembrane domain (Δ27 mutant; exon 11). This suggests that masitinib will be effective for the treatment of diseases linked to activating mutations in KIT, which includes mastocytosis, GIST, and canine mast cell tumours [6]. Furthermore, exon 11 mutants, which appear to be the most common type of KIT mutation in these diseases, were more sensitive to masitinib (IC_50_ = 3 to 20 nM) than the wild-type receptor (IC_50_ = 150 nM). In support of this, we found that mastocytoma cell lines carrying KIT juxtamembrane mutants had IC_50_ values for masitinib between 10 and 30 nM, whereas in murine primary BMMCs expressing wild-type KIT, the IC_50_ for masitinib was 200 nM. This higher sensitivity of juxtamembrane mutants than the wild-type receptor has also been reported for imatinib [28], [29].

Masitinib was a potent inhibitor of mutant PDGFR α and β receptors found in GIST and Chronic Myelomonocytic Leukaemia, respectively. Interestingly, masitinib is also very active against the protein FIP1L1-PDGFRα, which is generated from an internal deletion of chromosome 4 and is responsible for the induction of hypereosinophilic syndrome [30]. Masitinib therefore may be useful for the treatment of tumours involving mutant PDGF receptors.

Our studies also showed that masitinib is active *in vivo*. Intraperitoneal or oral administration of masitinib inhibited tumour growth in mice with subcutaneous grafts of Ba/F3 cells expressing the Δ27 KIT mutant. Furthermore, in an intraperitoneal model, masitinib significantly enhanced survival with no indication of general toxicity, as indicated by a lack of weight loss at the administered doses. These results demonstrate that masitinib is orally bioavailable and that it is effective at inhibiting tumour growth *in vivo*. This agrees with our phase 3 study in dogs showing that orally administered masitinib is safe and effective for the treatment of nonresectable or recurrent grade 2 or 3 nonmetastatic mast cell tumours [31].

In conclusion, our results show that masitinib is a potent and selective inhibitor of the KIT TK. Moreover, it appears to have higher affinity and selectivity *in vitro* than other TK inhibitors and does not inhibit kinases that are linked to toxic effects. Masitinib also potently inhibits recombinant PDGFR, the intracellular kinase Lyn, and, to a lesser extent, FGFR3. Additionally, masitinib was active and orally bioavailable. Thus, we anticipate that masitinib will be effective for the treatment of KIT and PDGFR-dependent diseases, which include various cancer and inflammatory diseases, and that it will have a better safety profile, especially regarding cardiotoxicity, than other KIT inhibitors.

## Materials and Methods

### Drug product

Masitinib was identified using a medicinal chemical approach to improve the selectivity of the phenylaminopyrimidine class of TK inhibitors [8], [9]. The chemical name is 4-(4-methylpiperazin-1-ylmethyl)-N-[4-methyl-3-(4-pyridin-3ylthiazol-2-ylamino) phenyl]benzamide-mesylate methane sulfonic acid salt, and the chemical formula is C_28_H_30_N_6_OS·CH_4_O_3_S (Figure 1A). Masitinib used in these studies was synthesised by either AB Science, S.A. (France), Archemis (Decines Charpieu, (France), Syngene (Bangalore, India) or by Prestwick Chemical, Inc. (France); for detailed procedure refer to patent WO/2008/098949. Its chemical structure was confirmed by nuclear magnetic resonance, mass spectrometry, ultraviolet and infrared spectrometry, and elemental analysis. Masitinib is practically insoluble in 0.1 M NaOH and n-hexane, slightly soluble in ethanol and propylene glycol, soluble in water, and freely soluble in 0.1 M HCl and dimethylsulfoxide. The compound, a white powder, was dissolved as a 10 or 20 mM stock solution in dimethylsulfoxide and stored at −80°C. Fresh dilutions of masitinib were made for each experiment. The imatinib used in this study was purchased from Sequoia Research (UK).

### *In vitro* assays with recombinant protein kinases

Full details for the generation of recombinant human KIT intracellular domain and other protein kinases (including Lyn, platelet derived growth factor receptor β, epidermal growth factor receptor, fibroblast growth factor receptor 1, Src, HCK, PYK, FES, Btk, Bmx, c-Ret, c-Fms, Syk, and c-Met) are provided in the Supplemental Methods (see Supporting Information; Methods S1). Experiments on ABL1, Akt1, protein kinase C-α, insulin-like growth factor receptor 1, and Pim1 were carried out by Proqinase (Germany). All other recombinant protein kinases were performed in-house using an enzyme-linked immunoassay; experimental details are provided in the Supplemental Methods (see Supporting Information; Methods S1).

### *In vitro* assays in intact cells

Ba/F3 cells [28], [32] were grown at 37°C in Roswell Park Memorial Institute medium (RPMI) 10 (RPMI 1640 with L-glutamine, supplemented with 100 units/ml penicillin, 100 µg/ml streptomycin, and 10% heat-inactivated foetal calf serum). The generation of Ba/F3 cells expressing wild-type or mutant (e.g. D^816^V and V^559^D) murine and human KIT has been previously described [19], [32]. All cells were analysed and sorted by FACS for cell surface expression of human KIT using MAB332, a mouse anti-KIT monoclonal antibody (R&D Systems Europe, France), and for murine KIT using ACK2, a rat anti-KIT monoclonal antibody (Clinisciences SA, France). Cells expressing the constitutively activated mutant forms of KIT mutant were selected according to their ability to proliferate in the absence of IL-3.

For the assay of Ba/F3 cell proliferation, microtitre plates were seeded with a total of 10^4^ cells/well in 100 µl of RPMI 1640 medium with 10% foetal bovine serum at 37°C. These were supplemented, or not, with either 0.1% conditioned medium from X63-IL-3 cells or 250 ng/ml murine SCF. The murine SCF, which activates KIT, was purified from the conditioned medium of SCF-producing CHO cells (gift of S. Lyman, Immunex). Cells were grown for 48 hours at 37°C and then incubated with 10 µl/well of WST-1 reagent (Roche Applied Science, France) for 3 hours at 37°C. The amount of formazan dye formed was quantified by its absorbance at 450 nm using a scanning multiwell spectrophotometer (MultiSkan MS, Thermo-LabSystems, France). A blank well without cells was used as a background control for the spectrophotometer and all assays were performed in triplicate.

Apoptotic and dead cells were detected using annexin V-phycoerythrin and 7-amino-actinomycin D via FACScan (Becton Dickinson, USA), according to the manufacturer's instructions (BD Biosciences Pharmingen, France). Full details for the analysis of tyrosine phosphorylation in intact cells are provided in the Supplemental Methods. Western blotting was performed using one of the following primary antibodies: for KIT, 1∶1000 dilution of a polyclonal rabbit anti-KIT antibody (Cell Signalling Technology, France); for PDGFR-α 0.2 µg/ml anti-PDGFR-α antibody sc-338 (Ozyme, France); for phosphotyrosine, using 1∶1000 anti-phosphotyrosine antibody 4G10 (Cell Signalling Technology). These were followed by 1∶10,000 horseradish peroxidase-conjugated anti-rabbit antibody (Jackson Laboratory, USA) or 1∶20,000 horseradish peroxidase-conjugated anti-mouse antibody (Dako-France SAS, France). Immunoreactive bands were detected using enhanced chemiluminescent reagents (Pierce, USA).

Assessment of the effect of masitinib and imatinib on human mast cell degranulation response and cytokine production (TNF-α release), was performed on CBMC produced by long-term culture of CD34+ progenitors purified from normal cord-blood, as described previously by Royer *et al* [33] (see Supporting Information; Methods S1). Cultured cells were harvested, washed in complete IMDM medium, and incubated for 1 hour in various concentrations of masitinib or imatinib. Assays of β-hexosaminidase release and TNF-α release were made by stimulating the CBMC with 1 µg/ml of goat anti-human IgE (Vector Laboratories) for 30 minutes or 4 hours, respectively. β-hexosaminidase was measured in the supernatant and in the sonicated cell pellets and its net release calculated [34]. For TNF-α determination, the cell-free supernatants were collected by centrifugation and frozen at −80°C until determination of mediator content by the use of a specific ELISA kit according to manufacturer's instructions (Coulter, France). All assays were performed in duplicate and counts were repeated twice for each well. Results were expressed in percentage of inhibition of β-hexosaminidase release and of TNF-α release relative to the stimulated untreated CBMC, (i.e. 100% of stimulation).

Migration of murine BMMCs was evaluated using a transwell migration assay [35]. Briefly, 2.5×10^5^ unstarved mast cells in 100 µL of chemotaxis buffer (RPMI 1640, 0.5% bovine serum albumin [BSA], 1% antibiotics) were loaded onto each transwell filter (8 µm pore, 24-well cell clusters; Becton Dickinson, USA). Filters were then placed in wells containing 600 µL of chemotaxis buffer supplemented with or without 10 ng/mL of rmSCF, for stimulated or unstimulated BMMCs, respectively. After 4 hours incubation at 37°C in 5% CO_2_, cells from the bottom chamber were resuspended and counted using a FACS Scan over 20 seconds. All assays were performed in triplicate and counts were repeated twice for each well. For tyrosine kinase inhibitor treatment, 1×10^7^ mast cells were pretreated for 1.5 hours at 37°C in complete medium (OPTI MEM, 10% foetal calf serum (FCS), 1% antibiotics and 2-mercaptoethanol 5×10^−5^ M, 10 ng/ml rIL3) either with 1 µM of inhibitor or an equivalent volume of DMSO.

### Molecular modelling

X-ray coordinates of the STI571/ABL (1IEP.pdb) and STI571/KIT (1T46.pdb) X-ray structures were taken from the Protein Databank and used in combination with our in-house docking program, ParaDocks, and the X-Score of Wang et al. [36] to dock masitinib into ABL and KIT. Figures were prepared with PyMOL version 1.00 (DeLano Scientific).

### *In vivo* assays with Ba/F3 Δ27 tumour model

Female MBRI Nu/Nu mice (7 weeks old) (Janvier, France) were housed under specific pathogen-free conditions at 20±1°C with a 12 hours light/12 hours dark cycle and *ad libitum* access to food and filtered water. The mice were allowed to acclimatise to the study conditions for 10 to 20 days prior to experiments. All animal experiments were performed according to Centre national de la recherche scientifique (CNRS) ethical guidelines of animal experimentation [37]. The animal care unit SCEA (Institut Gustave Roussy, Villejuif, France) is authorised by the French Ministries of Agriculture and Research (Agreement N° C94-116). The Δ27-expressing Ba/F3 cells were grown in RPMI 1640 medium supplemented with glutamax-1 (Gibco BRL, USA) and 10% foetal bovine serum (Gibco BRL, USA) at 37°C in a humidified atmosphere containing 5% CO_2_. The cells were centrifuged and resuspended at 5×10^6^ or 7.5×10^6^ cells/ml in phosphate-buffered saline. Mice were treated with 5 Gy of gamma radiation and after 24 hours they were injected in the right flank with 1.5×10^6^ Δ27 Ba/F3 cells. When tumour growth had reached the desired size, mice were allocated into treatment groups ensuring that there was no statistical difference between each group's mean body weight and tumour volume. For all animals, body weight was measured on the day of injection and every 5 days thereafter, with the tumour's size measured via callipers every 5 days during the treatment period for estimation of tumour volume. During the predose period and for 2 weeks post-treatment, the animals were checked for mortality or signs of morbidity once a day, increasing to twice a day checks during the treatment period.

### Statistical analysis

Assays for the *in vitro* effect of masitinib on the activity of protein kinases were performed as three independent experiments (each in duplicate), with results presented using descriptive statistics. Masitinib's effect on tumour growth was expressed in terms of estimated tumour volume = [length×width^2^]/2. Survival in the *in vivo* mouse studies was assessed by Kaplan-Meier analysis using GraphPad Prism (GraphPad Software, Inc. USA) with comparison of survival curves performed by the logrank Mantel-Cox test. The appropriate Wilcoxon or Kruskall-Wallis tests were used for group comparison of tumour volumes and BMMC migration.

## Supporting Information

Methods S1 Supplemental Methods
(0.06 MB DOC)

Table S1 Effect of imatinib on selected recombinant protein kinases.
(0.04 MB DOC)
