# Supplementary material for: Masitinib (AB1010), a Potent and Selective Tyrosine Kinase Inhibitor Targeting KIT
Source: PLoS One. 2009 Sep 30;4(9):e7258. doi: 10.1371/journal.pone.0007258 (PMC2746281; doi:10.1371/journal.pone.0007258)
Supplement: Methods S1 — Supplemental Methods (0.06 MB DOC) [file pone.0007258.s001.doc]

**Supplemental Methods**

*Generation of recombinant human KIT intracellular domain.*

The IMAGE cDNA clone encoding the full-length human KIT protein (Genbank Accession number BC071593) was purchased from Openbiosystems (Biovalley, France). The fragment encoding amino acids 567 to 976 fused to a *Bam*HI site and a hexahistidine tag at its 5’-end and to a *Xba*I site at its 3’-end was amplified by PCR with the following primers: forward, 5’‑GGATCCATGGGTCACCACCATCACCACCATGTACCAAATTATGTTTACATA‑3’ (*Bam*HI site underlined); reverse, 5’‑TCTAGACTCGAGTCAGACATCGTCGTGCACAAGCAGAG‑3’ (*Xba*I site underlined). The PCR product was cloned in the pGEMT-easy vector and sequenced on both strands using the BigDye terminator v3.1 cycle sequencing kit on a 3100 automated sequencing system (Applied Biosystems, Foster City, CA). The D^816^V (GAC→GTC) mutant was generated from the wild-type matrix cloned in pGEM-T-easy with the Quickchange site-directed mutagenesis kit (Stratagene, USA) according to manufacturer’s instructions using the following oligonucleotides: forward, 5’‑TAGCCAGAGTCATCAAGAAT‑3’ (mutation underlined); reverse, 5’‑ATTCTTGATGACTCTGGCTA‑3’ (mutation underlined). The appropriate cDNA inserts (wild-type and D^816^V) were excised from pGEMT-easy using *BamH*I and *Xba*I (New England Biolabs, UK) and cloned into the baculovirus expression vector pVL1393 at the same sites. Sf9 cells (Invitrogen, USA) were cultured in Grace’s insect medium (Invitrogen, USA) supplemented with 2 mM L-glutamine (Invitrogen, USA), 0.2 µg/ml streptomycin, and 200 international untis/ml penicillin G and were transfected with the BaculoGold™ Transfection Kit (BD Biosciences Pharmingen France, Belgium) according to the manufacturer’s instructions. Freshly seeded insect cells (5 × 10^6^ cells per 10-cm plate) were infected with 500 µl of transfection supernatant in 15 ml of TNM-FH medium (kit component). The cells were incubated at 27°C for 6 to 7 days before harvesting the medium. The medium was used for another round of amplification to generate a high-titer stock solution. High Five™ cells (Invitrogen) were grown in 150 cm^2^ flasks in 25 ml of Express Five serum-free medium (Invitrogen) and infected 5 hours after 1:5 dilution with 300 µl of the high-titer stock solution. The cells were harvested by centrifugation, washed in cold phosphate-buffered saline, and resuspended in cold lysis buffer (500 mM NaCl, Complete™ Protease cocktail inhibitor mix [Roche Diagnostics, France], 1 mM sodium orthovanadate, 1% Triton X-100, and 40 mM Tris-HCl, pH 9.0) and lysed with a French press at 720 psi. The lysate was centrifuged at 4°C for 60 minutes at 50 000 × *g* and filtered through a 0.22-µm filter. The recombinant KIT protein was purified in one step by affinity chromatography on a HiTrap Chelating column (Amersham-Pharmacia Biotech). The activity of the purified fraction was confirmed using a tyrosine kinase assay (see “In vitro kinase assay of recombinant tyrosine kinases”), and the purity of the recombinant protein was assessed by sodium dodecyl sulfate-polyacrylamide gel electrophoresis followed by Coomassie blue R-250 staining as well as by Western blotting with an antibody to hexahistidine (Qiagen S.A., France).

*Generation of recombinant human PDGF receptor β, Lyn, EGF receptor, fibroblast growth factor receptor 1, c-Src, Hck, Btk, Bmx, Fes, Ptk2, c-Ret, c-Fms, Syk, and c-Met.*

The sequences encoding PDGF receptor β (residues 554–1106; GenBank accession no. BC032224), Lyn B (2–583; accession no. BC059394), fibroblast growth factor receptor 1 (572–959; accession no. BC018128), Src (2–536; accession no. BC011566), Btk (2–659; accession no. BC109079), Bmx (2–675; accession no. BC016652), c-Ret (713–1114; accession no. BC004257), c-Fms (538–972; BC047531), Syk (2–612; BC011399), Hck (2-526; accession no. BC014435), Fes (2-822; accession no. BC035357), PYK2 (2-1006; accession no. BC042599) and c-Met (949–1398; accession no. lifeseq3489592) were amplified by PCR from IMAGE cDNA clones purchased from Openbiosystems. The cDNA encoding the EGF receptor (664–1210) was kindly provided by Dr. D. Birnbaum, Marseille Cancer Institute. Restriction sites for cloning were added with the following oligonucleotides using Phusion DNA polymerase (Ozyme, France): PDGF receptor β 5’ (*Sal*I), GTCGACGTGCTTTGGCAGAAGAAGCC; PDGF receptor β 3’(*EcoR*I), GAATTCCTACAGGAAGCTATCCTC; LynB 5’ (*Sal*I), GTCGACGTGGGATGTATAAAATC; LynB 3’ (*EcoR*I), GAATTCCTAAGGCTGCTGCTGG; fibroblast growth factor receptor 1 5’ (*Sal*I), GTCGACAGTGCATCCATGAACTCTGG; fibroblast growth factor receptor 1 3’ (*EcoR*I), GAATTCCAGTCAGCGGCGTTTGAG; Src 5’ (*Sal*I), GTCGACGGTAGCAACAAGAGCAAG; Src 3’ (*EcoR*I), GAATTCCTTCCATGCCACCAAAGAG; EGF receptor 5’ (*Sal*I), GTCGACTTCATGCGAAGGCGCC; EGF receptor 3’ (*Hind*III), GTCGACGGGTGCATGAAGTCCAAG; HCK 5’ (*Sal*I),

GAATTCTCATGGCTGCTGTTGGTAC; HCK 3’ (*EcoRI*),

GTCGACGGCTTCTCTTCTGAGCTG, FES 5’ (*Sal*I),

GAATTCTCACCGATGCCGCTTTCG, FES 3’ (*EcoRI*),

GTCGACTCTGGGGTGTCCGAGCCCCTG, PYK2 5’ (*Sal*I),

GAATTCTCACTCTGCAGGTGGTGTGGC, PYKé 3’(*EcoRI*), AAGCTTGTCATGCTCCAATAAATTC; c-Ret 5’ (*Sal*I), GTCGACGAGGATCCAAAGTGGG; c-Ret 3’ (*Hind*III), AAGCTTCTAGAATCTAGTAAATGCATG; Btk 5’ (*Sal*I), GTCGACGCCGCAGTGATTCTGGAGAG; Btk 3’ (*Spe*I), actagttcaggattcttcatccatg; Bmx 5’ (*Sal*I), GTCGACGATACAAAATCTATTCTAG; Bmx 3’ (*Sma*I), CCCGGGTCAATGCTTGTCTTTTTCC; c-Met 5’ (*Sal*I), GTCGACXGGGTTTTTCCTGTGGCTG; c-Met 3’ (*Xba*I), TCTAGACACTATGATGTCTCCCAG; Syk 5’ (*Sal*I), GTCGACGCCAGCAGCGGCATGGC; Syk 3’ (*Xba*I), ctcgagttagttcaccacgtcatag; c-Fms 5’ (*EcoR*I), GAATTCCTACAAGTATAAGCAGAAGCC; c-Fms 3’ (*Xba*I),TCTAGACAACTCCTCAGCAGAACTG. PCR products were inserted in pGEM-T Easy vector (Promega France SARL, France) and kinase inserts were fully sequenced with SP6 and T7 primers before being cloned in the pDNR1r vector (**Takara Bio Europe/Clontech**, France) at the corresponding restriction sites.

Baculovirus expression vectors were generated by CRE/LoxP recombination between the pDN1r donor vectors and pLP-BacPAK9-6xHN acceptor vector (**Takara Bio Europe/Clontech**, France) according to the manufacturer’s instructions. Sf9 cells were cultured in Grace’s insect medium supplemented with 2 mM L-glutamine, 0.2 µg/ml streptomycin and 200 UI/ml penicillin G. Recombinant baculoviruses were generated by co-transfection cells with pLP-BacPAK9-6xHN expression vectors and BacPAK6 DNA (**Takara Bio Europe/Clontech**, France) according to the manufacturer’s instructions. After the transfection, the recombinant baculoviruses were amplified three to four times to obtain a high-titer stock solution. The titer was determinated by enzyme-linked immunosorbent assay using the BacPAK Rapid baculovirus titer kit (**Takara Bio Europe/Clontech**, France) according to the manufacturer’s instructions.

*In vitro enzyme-linked immunoassay with recombinant protein kinases.*

A 96-well microtitre plate (Immulon 2HB; ThermoLifeScience, Finland) was coated overnight with 0.25 mg/ml poly(Glu,Tyr 4:1), rinsed twice with 250 µl of washing buffer (10 mM phosphate-buffered saline [pH 7.4] and 0.05% Tween 20) and dried for 2 hours at room temperature. Assays were performed at room temperature with a final volume of 50 µl in kinase buffer (10 mM MgCl_2_, 1 mM MnCl_2_, 1 mM sodium orthovanadate, 20 mM HEPES, pH 7.8) containing ATP at a concentration of at least twice the K_m_ for each enzyme and an appropriate amount of recombinant enzyme to ensure a linear reaction rate. Reactions were initiated upon introduction of the enzyme and terminated with the addition of one reaction volume (50 μl) of 100 mM EDTA per 5 M urea mix. Plates were washed three times and incubated with 1:30,000 horseradish peroxidase-conjugated anti-phosphotyrosine monoclonal antibody (Sigma-Aldrich, France), then washed three times and incubated with tetramethylbenzidine. The final reaction product was quantified by spectrophotometry at 450 nm.

*Method for analysis of tyrosine phosphorylation in intact cells.*

For analysis of tyrosine phosphorylation in intact cells, the Ba/F3 cells (5×10^6^) expressing KIT or PDGFR-α were serum-starved for 1 to 3 hours in a RPMI 1640 medium. Cells expressing wild-type KIT were treated for 5 minutes at 37°C with or without 250 ng/ml recombinant murine SCF (gift of S. Lyman, Immunex). Cells expressing PDGFR-α were treated for 5 minutes at 37°C with or without 10 ng/ml PDGF-BB (Peprotech France, France). The cells were then placed on ice and washed with ice-cold phosphate-buffered saline, lysed in 500 µl of ice-cold HNTG buffer (50 mM HEPES, pH 7, 50 mM NaF, 1 mM EGTA, 150 mM NaCl, 1% Triton X-100, 10% glycerol, and 1.5 mM mgCl_2_) containing a mixture of protease inhibitors (Roche Applied Science, France) and 100 µM Na_3_VO_4_. Cell lysates were mixed overnight at 4°C with 50 µl of a 20% slurry of protein G-Sepharose (Amersham Biosciences Europe GmbH, France) in phosphate-buffered saline and 2 µg of purified rabbit anti-KIT immune serum directed against the KIT cytoplasmic tail (BD Biosciences, France) or anti-PDGFR-α antibody sc-338 (Ozyme, France). The beads were washed in cold HTNG and resuspended in 20 µl of 2X electrophoresis sample buffer. The immunoprecipitated proteins were then separated by sodium dodecyl sulfate-polyacrylamide gel electrophoresis on an 8% acrylamide gel. Proteins were electrophoretically transferred onto a polyvinylidene difluoride membrane (Millipore SAS, France), which were blocked in Tris-buffered saline containing 0.05% Tween-20 and 5% bovine serum albumin for 30 to 45 minutes at room temperature.

*Generation of human cord-blood-derived mast cells.*

Human CBMC were produced by long-term culture of CD34+ progenitors purified from normal cord-blood [33]. CD34+ cells, purified from light density cord blood cells by the use of a specific immunoaffinity system (Miltenyi Biotech, France) were cultured at a starting density of 10^5^ cells/ml in a complete serum-free medium made by Iscove Dulbecco’s Modified Medium (IMDM) (Bio Whittaker, Belgium) supplemented with L-glutamine (Sigma), penicillin, streptomycin (Gibco, France), 1% bovine serum albumin (Boehringer Manheim, France), insulin (10 µg/ml) (Sigma), transferrin (5 µg/ml) (Sigma), 100 ng/ml of rmSCF **(R&D Systems Europe,** France) and 50 ng/ml rhIL-6 (**R&D Systems Europe**, France). At the beginning of the culture, rhIL-3 (1 ng/ml) (**R&D Systems Europe,** France) was also added to induce maximal expansion of haematopoietic progenitors. After 3 days the IL-3 was removed and 4 days later cells were transferred to a complete serum-free medium containing rmSCF at 100 ng/ml, which was renewed every 7 days thereafter. After 8 weeks in culture ≥99% of the cells were identified as being mast cells by May-Grünwald-Giemsa staining of cytocentrifuged samples. CBMCs (1x10^6^cells/ml) were subsequently incubated over 4 days at 37°C with 2.5 µg/ml of human myeloma IgE (Vector Laboratories, France) and 20 ng/ml of IL-4 (**R&D Systems Europe**, France) to induce optimal expression of the high affinity receptor for IgE (FcεRI).
