# Supplementary material for: Masitinib (AB1010), a Potent and Selective Tyrosine Kinase Inhibitor Targeting KIT
Source: PLoS One. 2009 Sep 30;4(9):e7258. doi: 10.1371/journal.pone.0007258 (PMC2746281; doi:10.1371/journal.pone.0007258)
Supplement: Table S1 — Effect of imatinib on selected recombinant protein kinases. (0.04 MB DOC) [file pone.0007258.s002.doc]

**Table S1. Effect of imatinib on selected recombinant protein kinases.**

| **Protein kinase** | **IC_50_ (μM)** |
| --- | --- |
| KIT | 0.47 ± 0.12 (n=2) |
| PDGF receptor β | 0.44 ± 0.12 (n=3) |
| PDGF receptor α | 0.4 (n=1) |
| ABL1 | 0.27 ± 0.13 (n=3) |
| Lyn B | 2.2 ± 1.0 (n=5) |
| Src | 3.6 ± 1.2 (n=3) |

Recombinant tyrosine kinase assays were performed using an ELISA-based assay with poly(Glu,Tyr, 4:1) as a substrate. All protein kinases were human versions. Results are the means ± standard deviations.
